# Supplementary material for: Vaccine-elicited IL-1R signaling results in Th17 TRM-mediated immunity
Source: Commun Biol. 2024 Apr 9;7:433. doi: 10.1038/s42003-024-06138-0 (PMC11003962; doi:10.1038/s42003-024-06138-0)
Supplement: Supplementary file 2 — Supplementary Information [file 42003_2024_6138_MOESM2_ESM.pdf]

Supplementary Information

Vaccine-elicited IL-1R signaling results in Th17 TRM-mediated immunity

Joseph P. Hoffmann[1], Akhilesh Srivastava[1], Haoran Yang[1], Naoki Iwanaga[1,2], T. Parks Remcho[1], Jenny L. Hewes[1], Rayshma Sharoff[1], Kejing Song[1], Elizabeth B. Norton[3], Jay K. Kolls[1], and Janet E. McCombs[1*]

[1]Center for Translational Research in Infection and Inflammation Tulane University School of Medicine, New Orleans, Louisiana, USA;

[2]Department of Respiratory Medicine, Nagasaki University Hospital, Nagasaki, Japan; and

[3]Department of Immunology and Microbiology, Tulane University School of Medicine, New Orleans, Louisiana, USA.

*Corresponding author: J.E. McCombs, jmccombs@tulane.edu

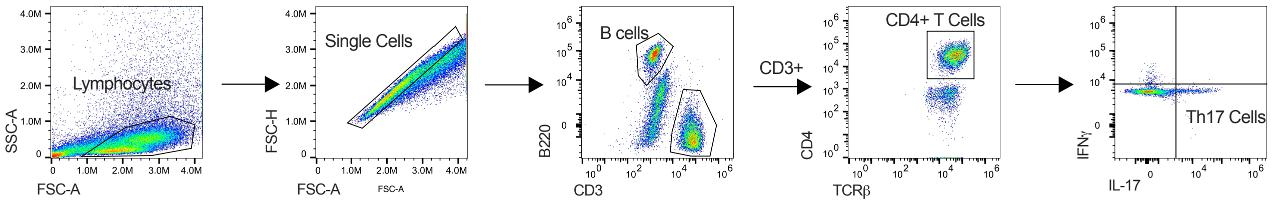

Supplementary Figure 1: Gating Strategy to detect lung CD4 T cells, B cells, and Th17 cells. Adaptive immune cells were identified first by using forward and side scatter to gate for lymphocytes and single cells. From there cells were gated as CD3+B220- or CD3-B220+ (B cells). CD3+B220- cells that were positive for CD4 and TCR-β were identified as CD4+ T cells. From this population, Th17 cells were identified based on positive staining for IL-17A and negative staining for IFNγ.

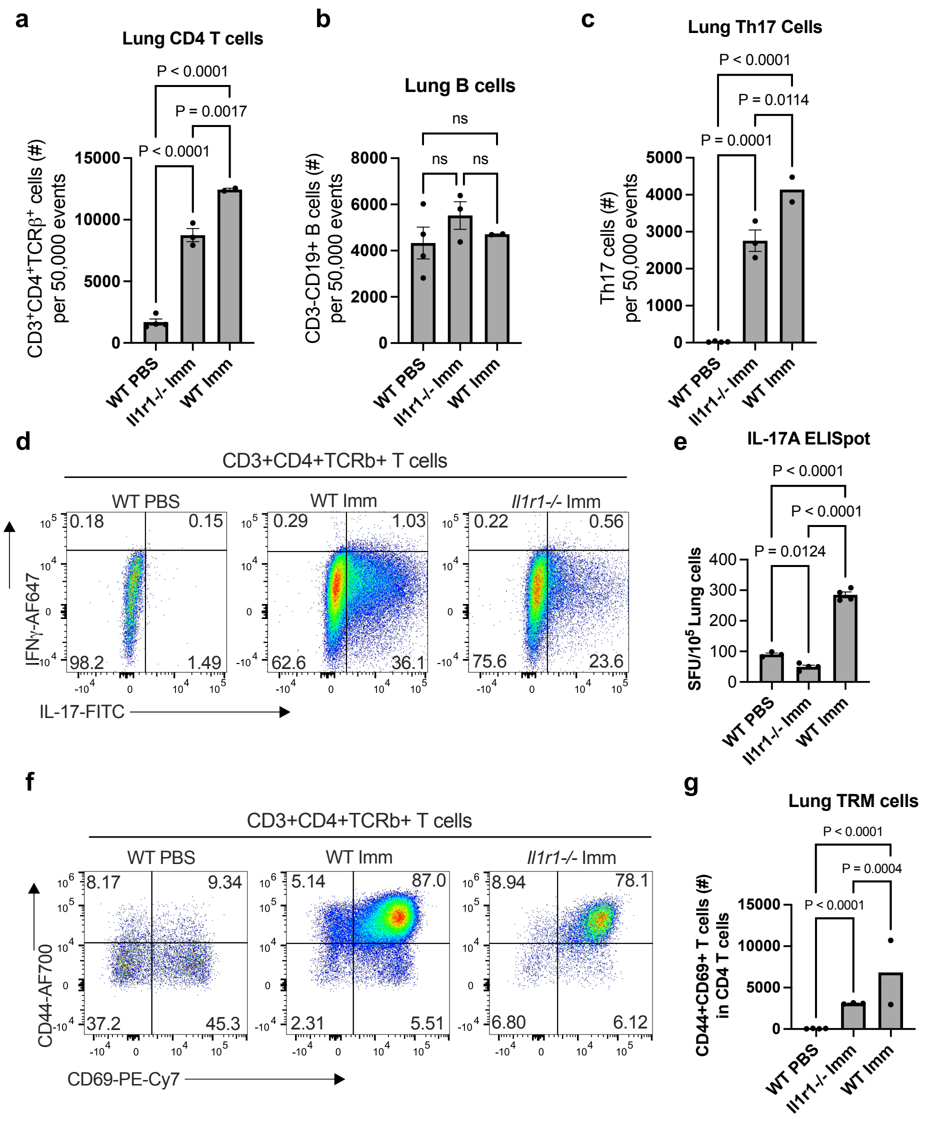

Supplementary Figure 2: IL-1 signaling-mediated generation of lung resident Th17 cells is dependent on OmpX + LTA1 immunization. Quantification of (**a**) CD4 T cells, (**b**) B cells, and (**c**) Th17 cells in the lungs of vaccinated and unvaccinated wild-type and *Il1r1*$^{-/-}$ mice as measured in flow cytometry. (**d**) Representative flow cytometry dot plots for determining lung Th17 cells (CD3^{+}, CD4^{+}, TCR-β^{+}, IL-17A^{+}) in immunized and unimmunized wildtype and *Il1r1*$^{-/-}$ mice. (**e**) ELISpot results measuring IL-17A secreting cells after overnight stimulation with OmpX. Data are displayed as spot counts (SFU) per 10^5 plated cells from single cell lung suspensions. (**f**) Representative flow cytometry dot plots for determining lung TRM cells (CD3^{+}, CD4^{+}, TCR-β^{+}, CD44^{+}, CD69^{+}) in unimmunized wildtype and immunized wildtype and *Il1r1*$^{-/-}$ mice. (**g**) Quantification of the total number of lung TRM cells in the lungs of vaccinated and unvaccinated wild-type and *Il1r1-/-* mice as measured by flow cytometry. Antibodies for flow were as follows: BV750 rat anti-mouse CD19 (1D3, BD, Cat #747332), APC rat anti-mouse CD3e (clone 17A2, Biolegend, Cat #100236), PerCP-Cy5.5 rat anti-mouse CD4 (clone GK1.5, Biolegend, Cat #100433), PE-Cy5 hamster anti-mouse TCRβ (clone H57-597, BD Biosciences, Cat# 553173), FITC rat anti-mouse IL-17A (clone TC11-18H10.1, Biolegend, Cat #506907), AlexaFluor 647 rat anti-mouse IFNγ (clone XMG1.2, Biolegend, Cat #505814), PE-Cy7 hamster anti-mouse CD69 (clone H1.2F3, eBioscience, Cat #25-0691-82), and AlexaFluor 700 rat anti-mouse/human CD44 (clone IM7, Biolegend, Cat #103026). Data are represented as mean +/- SEM (n = 4 mice, WT PBS; n = 3 mice, *Il1r1-/-* Imm, n = 2 mice, WT Imm). Statistical differences were determined using a one-way ANOVA followed by Tukey's multiple comparison test.

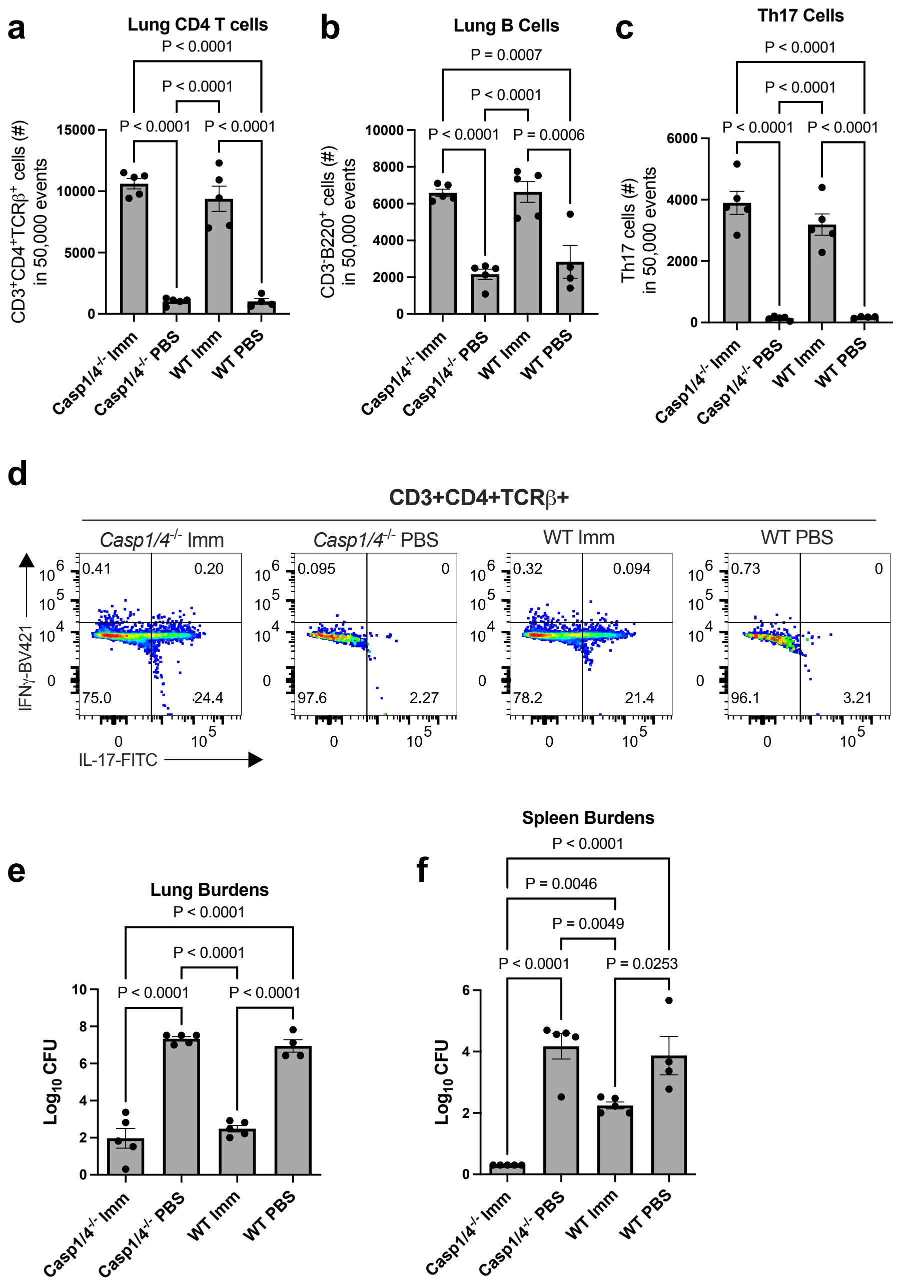

Supplementary Figure 3: Caspase1/4$^{-/-}$ mice have no change in protection and adaptive immune populations following immunization. *Casp1/4$^{-/-}$* mice and wildtype C57Bl/6 mice were vaccinated via oropharyngeal aspiration with OmpX + LTA1 or PBS. One week following immunization, mice were challenged with *K. pneumoniae* as described. Quantification of (**a**) CD4 T cells, (**b**) B cells, and (**c**) Th17 cells in the lungs of vaccinated and unvaccinated WT and *Casp1/4$^{-/-}$* mice, as measured in flow cytometry. (**d**) Representative flow plots of Th17 cells (CD3^{+}, CD4^{+}, TCR-β^{+}, IL-17A^{+}) in each group. There was no difference in tested adaptive immune cell populations, including Th17 cell numbers, between vaccinated *Casp1/4$^{-/-}$* and wildtype mice. This is similar to observations in Figure 3. **e-f**. Log transformed bacterial burdens in the lungs (e) and spleen (f) 24h post challenge in each group. Presence of caspase 1 and 4 and its downstream products IL-1β and IL-1α to a lesser extent had no impact on protection from challenge. Data are represented as mean +/- SEM (n = 4 mice, WT PBS; n = 5 mice, all other groups). Statistical differences were determined using a one-way ANOVA followed by Tukey's multiple comparison test. *Imm*, immunized.

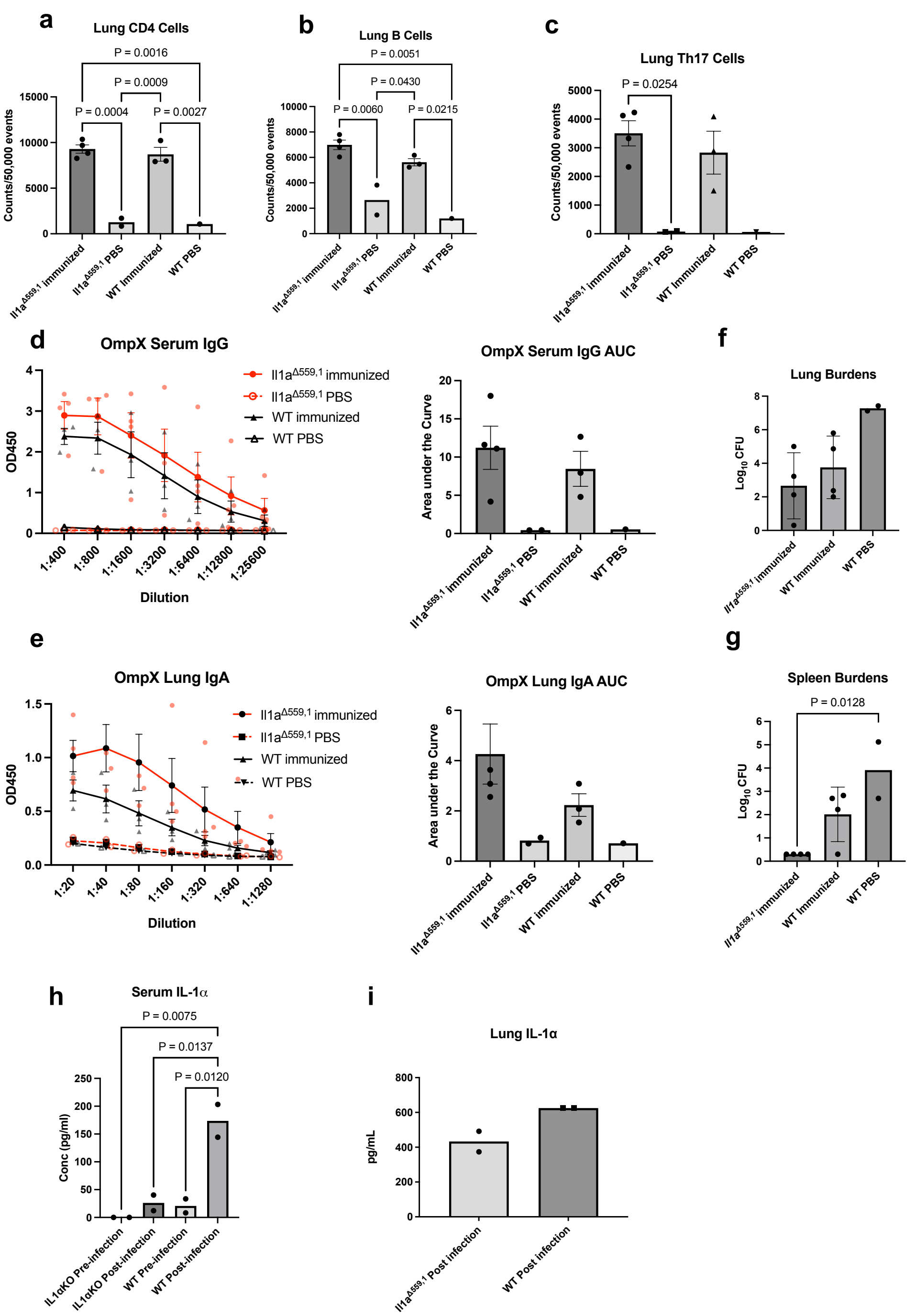

Supplementary Figure 4: *$Il1a^{\Delta559,1}$*mice have no change in protection following vaccination. *$Il1a^{\Delta559,1}$* and wildtype C57Bl/6 mice were vaccinated via oropharyngeal aspiration with OmpX + LTA1 or PBS. One week following immunization mice were challenged with *K. pneumoniae* as described. **a-c.** Quantification of CD4 T cell (a), B cell (b), and Th17 cell (c) populations in the lungs from each group (n = 1 mouse, WT PBS; n = 2 mice, $Il1a^{\Delta559,1}$, PBS; n = 3 mice, WT Imm; n = 4 mice, $Il1a^{\Delta559,1}$, Imm). There was no difference in tested adaptive immune cell populations despite reduced levels of IL-1α. (**d**) Anti-OmpX serum IgG and (**e**) lung homogenate IgA titers with their respective AUC analysis (n = 1 mouse, WT PBS; n = 2 mice, $Il1a^{\Delta559,1}$, PBS; n = 3 mice, WT Imm; n = 4 mice, $Il1a^{\Delta559,1}$, Imm). Decreased amounts of IL-1α did not impact antibody titers in vaccinated groups. **f-g**. Log transformed bacterial burdens in the lungs (f) and spleen (g) 24h post challenge in each group (n = 2 mice,

WT PBS; n = 4 mice, all other groups). Though there was high variability, immunized IL-1α hypomorph mice still had protection from challenge demonstrated by reduced bacterial burdens, particularly in the spleen. (**h)** Levels of IL-1α was measured in the serum of unvaccinated *Il1a*$^{\Delta 559,1}$and wildtype mice before (pre) and after (post) challenge. (**i**) IL-1α levels in the lung homogenate were taken from each group post infection (n=2 per group). IL-1α hypomorphs had similar levels of IL-1α in the lungs when compared to wildtype mice. This suggests that they may be a poor model for IL-1α depletion. Data are represented as mean +/- SEM. Statistical differences were determined using a one-way ANOVA followed by Tukey's multiple comparison test. *Imm*, immunized.

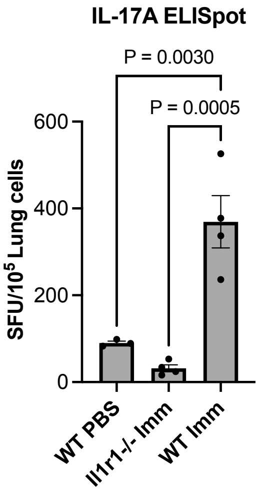

Supplementary Figure 5: IL-1 signaling-mediated generation of OmpX-specific lung resident Th17 cells is dependent on OmpX + IL-1α immunization. ELISpot results measuring IL-17A secreting cells after overnight stimulation with OmpX. Data are displayed as spot counts (SFU) per 10^5 plated cells from lung homogenate (n = 3 mice, WT PBS; n = 4 mice, all other groups). Data are represented as mean +/- SEM. Statistical differences were determined using a one-way ANOVA followed by Tukey's multiple comparison test. *Imm*, immunized.
