# Supplementary material for: Vaccine-elicited IL-1R signaling results in Th17 TRM-mediated immunity
Source: Commun Biol. 2024 Apr 9;7:433. doi: 10.1038/s42003-024-06138-0 (PMC11003962; doi:10.1038/s42003-024-06138-0)
Supplement: Supplementary file 3 — Description of Additional Supplementary Files [file 42003_2024_6138_MOESM3_ESM.pdf]

Description of Additional Supplementary Files

File name: Supplementary Data 1

Description: The source data behind the graphs in Figures 1-5 of the paper.
