# Supplementary material for: Vaccine-elicited IL-1R signaling results in Th17 TRM-mediated immunity
Source: Commun Biol. 2024 Apr 9;7:433. doi: 10.1038/s42003-024-06138-0 (PMC11003962; doi:10.1038/s42003-024-06138-0)
Supplement: Supplementary file 5 — Reporting Summary [file 42003_2024_6138_MOESM5_ESM.pdf]

nature portfolio

Corresponding author(s): Janet E. McCombs

Last updated by author(s): Mar 21, 2024

Reporting Summary

Nature Portfolio wishes to improve the reproducibility of the work that we publish. This form provides structure for consistency and transparency in reporting. For further information on Nature Portfolio policies, see our Editorial Policies and the Editorial Policy Checklist.

Statistics

For all statistical analyses, confirm that the following items are present in the figure legend, table legend, main text, or Methods section.

n/a	Confirmed	
☐	☒	The exact sample size (*n*) for each experimental group/condition, given as a discrete number and unit of measurement
☐	☒	A statement on whether measurements were taken from distinct samples or whether the same sample was measured repeatedly
☐	☒	The statistical test(s) used AND whether they are one- or two-sided<br>*Only common tests should be described solely by name; describe more complex techniques in the Methods section.*
☐	☒	A description of all covariates tested
☐	☒	A description of any assumptions or corrections, such as tests of normality and adjustment for multiple comparisons
☐	☒	A full description of the statistical parameters including central tendency (e.g. means) or other basic estimates (e.g. regression coefficient) AND variation (e.g. standard deviation) or associated estimates of uncertainty (e.g. confidence intervals)
☐	☒	For null hypothesis testing, the test statistic (e.g. *F*, *t*, *r*) with confidence intervals, effect sizes, degrees of freedom and *P* value noted<br>*Give P values as exact values whenever suitable.*
☒	☐	For Bayesian analysis, information on the choice of priors and Markov chain Monte Carlo settings
☒	☐	For hierarchical and complex designs, identification of the appropriate level for tests and full reporting of outcomes
☒	☐	Estimates of effect sizes (e.g. Cohen's *d*, Pearson's *r*), indicating how they were calculated

Our web collection on statistics for biologists contains articles on many of the points above.

Software and code

Policy information about availability of computer code

Data collection	NextSeq Software version 2.2.0.2<br>SpectroFlo (Cytek)<br>BioTek Gen5 Software (Agilent)<br>ImmunoSpot Software (Cellular Technology Ltd)
Data analysis	Cell Ranger version 2.1.1 (10X Genomics)<br>Loupe Cell Browser 6 (10X Genomics)<br>Seurat suite version 2.2.1<br>FlowJo software v9 and v10 (Tree Star)<br>LEGENDplex™ Data Analysis Software Suite (Qognit)<br>GraphPad Prism (GraphPad Software, LLC, v9.4.1)<br>ImmunoSpot Software (Cellular Technology Ltd)

For manuscripts utilizing custom algorithms or software that are central to the research but not yet described in published literature, software must be made available to editors and reviewers. We strongly encourage code deposition in a community repository (e.g. GitHub). See the Nature Portfolio guidelines for submitting code & software for further information.

Data

Policy information about availability of data

All manuscripts must include a data availability statement. This statement should provide the following information, where applicable:

- Accession codes, unique identifiers, or web links for publicly available datasets
- A description of any restrictions on data availability
- For clinical datasets or third party data, please ensure that the statement adheres to our policy

The sequencing data in Figure 1 have been deposited in the database Gene Expression Omnibus and can be accessed using accession numbers GSE178385 (scRNA-seq). All other data needed to evaluate the conclusions in the paper are present in the paper or the Supplementary Materials.

Research involving human participants, their data, or biological material

Policy information about studies with human participants or human data. See also policy information about sex, gender (identity/presentation), and sexual orientation and race, ethnicity and racism.

Reporting on sex and gender	Not relevant, no human studies involved in this research
Reporting on race, ethnicity, or other socially relevant groupings	Not relevant, no human studies involved in this research
Population characteristics	Not relevant, no human studies involved in this research
Recruitment	Not relevant, no human studies involved in this research
Ethics oversight	Not relevant, no human studies involved in this research

Note that full information on the approval of the study protocol must also be provided in the manuscript.

Field-specific reporting

Please select the one below that is the best fit for your research. If you are not sure, read the appropriate sections before making your selection.

☒ Life sciences ☐ Behavioural & social sciences ☐ Ecological, evolutionary & environmental sciences

For a reference copy of the document with all sections, see nature.com/documents/nr-reporting-summary-flat.pdf

Life sciences study design

All studies must disclose on these points even when the disclosure is negative.

Sample size	Sample sizes were chosen based on previous studies using this vaccine in mice (PMID: 34516780), suggesting 6-10 mice per group is sufficient to see statistical differences in vaccine studies.
Data exclusions	No data were excluded from analysis.
Replication	Experiments were performed in duplicate to verify results and results were replicated, unless noted otherwise. Data presented are pooled from all experiments performed.
Randomization	Mice were either purchased from Jax and randomly assigned to groups or bred in-house and randomly selected from litters.
Blinding	Investigators were not blinded during data collection or analysis. All data was processed using software (i.e. Graphpad, Prism v9) and reviewed and verified with other researchers.

Reporting for specific materials, systems and methods

We require information from authors about some types of materials, experimental systems and methods used in many studies. Here, indicate whether each material, system or method listed is relevant to your study. If you are not sure if a list item applies to your research, read the appropriate section before selecting a response.

Materials & experimental systems

n/a	Involved in the study
☐	☒ Antibodies
☒	☐ Eukaryotic cell lines
☒	☐ Palaeontology and archaeology
☐	☒ Animals and other organisms
☒	☐ Clinical data
☒	☐ Dual use research of concern
☒	☐ Plants

Methods

n/a	Involved in the study
☒	☐ ChIP-seq
☐	☒ Flow cytometry
☒	☐ MRI-based neuroimaging

Antibodies

Antibodies used

Rat Anti-Mouse CD16/CD32 Fc Block (clone 2.4G2, BD Biosciences, Cat# 553141), PE-Cy7 Rat Anti-Mouse CD4 (clone RM4-5, BD Biosciences, Cat# 561099), APC rat anti-mouse CD3e (clone 17A2, Biolegend, Cat#100236), PE-Cy5 hamster anti-mouse TCRβ (clone H57-597, BD Biosciences, Cat# 553173), FITC rat anti-mouse IL-17A (clone TC11-18H10.1, Biolegend, Cat# 506907), Brilliant Violet 421 rat anti-mouse IFNγ (clone XMG1.2, Biolegend, Cat# 505829), PE rat anti-mouse B220 (clone RA3-6B2, Biolegend, Cat# 103207), BV750 rat anti-mouse CD19 (1D3, BD, Cat #747332), PerCP-Cy5.5 rat anti-mouse CD4 (clone GK1.5, Biolegend, Cat # 100433), AlexaFluor 647 rat anti-mouse IFNg (clone XMG1.2, Biolegend, Cat #505814), PE-Cy7 hamster anti-mouse CD69 (clone H1.2F3, eBioscience, Cat #25-0691-82), AlexaFluor 700 rat anti-mouse/human CD44 (clone IM7, Biolegend, Cat #103026), anti-IL-1α (clone ALF-161, BioXCell, Cat #BE0243), anti-IL-1β (clone B122, BioXCell, Cat #BE0246), isotype control polyclonal Armenian hamster IgG (polyclonal, BioXCell, Cat #BE0091), goat anti-mouse IgG-HRP (polyclonal, Southern Biotech, Cat# 1036-05), goat anti-mouse IgA-HRP (polyclonal, Southern Biotech, Cat# 2050-05), anti mouse IL-17A antibody (clone 50101, R&D, #MAB721-100), biotinylated anti-mouse IL-17A antibody (polyclonal, R&D, #BAF421), PE hamster anti-mouse cd121a (clone JAMA-147, Biolegend, Cat# 113505), AlexaFluor 647 mouse anti-mouse CD45.2 (clone 104, BioLegend, Cat #109818), PE-Cy5 hamster anti-mouse CD3e (clone 145-2C11, BD, Cat #561825), BV480 rat anti-mouse CD19 (clone 1D3, Invitrogen, Cat #414-0193-82), PerCP-Cy5.5 rat anti-mouse CD4 (clone RM4-5, BioLegend, Cat #100540).

Validation

All antibodies were used as per the manufacturer recommendations. Antibodies for flow cytometry, ELISA, and ELISpot have been previously validated by our group (unpublished and PMID: 34516780). The anti-IL-1α and anti-IL-1β antibodies have been shown by the manufacturer to neutralize the bioactivity of both natural and recombinant protein. Control antibodies were used where indicated to eliminate effects of non-specific binding.

Animals and other research organisms

Policy information about studies involving animals; ARRIVE guidelines recommended for reporting animal research, and Sex and Gender in Research

Laboratory animals

Male mice aged 7 – 10 weeks on a C57Bl/6J background were used. Strains included wild-type, Il1r1-/- (Strain #:003245) , Casp1-/- (Strain #:032662) , Casp1/4-/- (Strain #:016621) , and Il1adelta559,1 (Strain #: 067031-JAX).

Wild animals

Study did not involve wild animals.

Reporting on sex

Findings only apply to male mice. This was a follow-up on our initial vaccine study using male mice. Preliminary data from our group indicate no sex difference in vaccine-mediated protection, suggesting similar results may be found in female mice.

Field-collected samples

Study did not involve field-collected samples.

Ethics oversight

Experimental procedures were conducted in accordance with protocols approved by Tulane University's Institutional Animal Care and Use Committee.

Note that full information on the approval of the study protocol must also be provided in the manuscript.

Flow Cytometry

Plots

Confirm that:

☒ The axis labels state the marker and fluorochrome used (e.g. CD4-FITC).

☒ The axis scales are clearly visible. Include numbers along axes only for bottom left plot of group (a 'group' is an analysis of identical markers).

☒ All plots are contour plots with outliers or pseudocolor plots.

☒ A numerical value for number of cells or percentage (with statistics) is provided.

Methodology

Sample preparation	The left lung of euthanized mice was collected in 700 µL sterile PBS and kept on ice for further processing. The PBS was decanted, and the tissue was minced manually with dissection scissors. Minced tissue was resuspending in 2 mL IMDM (Gibco) containing 2 mg/mL collagenase (Sigma-Aldrich) and 80 U/mL DNase1 (Sigma-Aldrich) and incubated at 37ºC with shaking at 233 rpm for 1 hour. Digested tissue was passed through a 70 µm cell strainer (Fisher) and red blood cells were removed using ACK lysis buffer (Gibco). Isolated cells were resuspended in 1 mL IMDM containing 10% FBS (Hyclone) and counted on a Cellometer for downstream applications.
Instrument	Cytek Aurora spectral flow cytometer
Software	For data collection: SpectroFlo (Cytek)<br>For data analysis: FlowJo software v9 and v10 (Tree Star)
Cell population abundance	In general, greater than 60% of CD3+ cells were CD4 T cells, ~40% of CD3- cells were determined to be B cells, and about 30% of CD4 T cells were determined to be Th17 (in wildtype animals). Numbers based off of flow cytometry dot-plots.
Gating strategy	Adaptive immune cells were identified first by using forward and side scatter to gate for lymphocytes and single cells. From there cells were gated as CD3+ or CD3- . CD3- cells were classified as B cells if they were positive for B220. CD3+ cells that were positive for CD4 and TCR-β were identified as CD4 T cells. From this population, Th17 cells were identified based on positive staining for IL-17A. Gating strategy is outlined in Supplementary Figure 1.

☒ Tick this box to confirm that a figure exemplifying the gating strategy is provided in the Supplementary Information.
